# Using the Satisfaction with Life Scale in People with Parkinson's Disease: A Validation Study in Different European Countries

**DOI:** 10.1155/2014/680659

**Published:** 2014-02-02

**Authors:** Ramona Lucas-Carrasco, Brenda L. Den Oudsten, Erhan Eser, Michael J. Power

**Affiliations:** ^1^Department of Methodology and Behavioural Sciences, University of Barcelona, Passeig Valld' Hebron 171, 08035 Barcelona, Spain; ^2^Cambridgeshire and Peterborough NHS Foundation Trust, Beechcroft, Fulbourn Hospital, Cambridge CB21 5EF, UK; ^3^Department of Medical Psychology, Center of Research on Psychology in Somatic Diseases (CoRPS), Tilburg University, Hilvarenbeekse Weg 60, 5022 GC Tilburg, The Netherlands; ^4^Department of Education and Research, St. Elisabeth Hospital, 5000 LE Tilburg, The Netherlands; ^5^Department of Public Health, Faculty of Medicine, Celal Bayar University, 45030 Manisa, Turkey; ^6^Section of Clinical Psychology, School of Health in Social Science, University of Edinburgh, Edinburgh EH8 9AG, UK; ^7^Department of Psychology, University of Tromsø, 9037 Tromsø, Norway

## Abstract

*Background*. Overall, people with chronic illnesses have lower life satisfaction compared to nonclinical populations. The objective of this international study was to examine the psychometric properties of the Satisfaction with Life Scale (SWLS) in patients with Parkinson's disease (PD). *Methods*. PD patients (*n* = 350) were recruited and interviewed at different specialized services in the United Kingdom, Spain, Czech Republic, Italy, and The Netherlands. A questionnaire set including a measure of life satisfaction, quality of life (QoL), self-reported health and disability status, and sociodemographic information was used. Acceptability, reliability, and validity were examined. *Results*. The internal consistency was good (**α** = 0.81). The scale structure was satisfactory (comparative fit index = 0.99; root mean square error of approximation = 0.08). The SWLS was able to discriminate between healthy and unhealthy, disabled and nondisabled, and those perceiving a more severe impact of the disability on their lives. Concurrent validity using multiple linear regression models confirmed associations between SWLS and QoL and age. *Conclusions*. This study is the first to report on the use of the SWLS in PD patients in different European countries. It is a useful tool in assessing satisfaction with life in PD patients through the continuum of care.

## 1. Introduction

Parkinson's disease (PD) is a common disease of unknown etiology in elderly people and one of the major causes of disability among elderly [1, 2]. In many cases, PD is associated with disabilities, not only with physical disability, but also with impairment and restrictions in emotional, cognitive, and social functioning [3], which might affect life satisfaction. The goal among people with chronic illnesses, most of which are associated with disability, is to promote and maintain subjective well being [4] and quality of life (QoL) [5, 6]. Subjective well being includes three distinct concepts: positive affect, negative affect, and life satisfaction [7]. The first two concepts, as their names imply, refer to an emotional or affective state, while life satisfaction is viewed as the cognitive element, the area dealing with a person's acquisition, processing, and use of knowledge, all of which act in concert to shape self-perceptions [4].

One of the scales widely used to appraise life satisfaction is the Satisfaction with Life Scale (SWLS) [8]. The psychometric properties of the SWLS have been examined in different nonclinical populations and less frequently in clinical samples [9]. Among clinical samples, the SWLS has been used in patients with chronic illnesses, such as mental illness [10], systemic lupus erythematosus [11], arthritis [12], and MS [13]; these studies have reported lower life satisfaction scores (slightly below average) compared to people without chronic illnesses. Only a few studies [12, 13] have examined the psychometric properties of the scale.

To the best of our knowledge, no studies were found investigating life satisfaction measured by the SWLS in PD patients. Therefore, this study aimed to examine the psychometric properties of the SWLS in PD patients in five European countries. In addition, the correlates associated with life satisfaction were examined.

## 2. Materials and Methods

### 2.1. Design and Procedure

A multicenter, cross-sectional study was employed. This study was part of the DISQOL Project funded by the European Commission Framework 6 Programme (number 513723). Detailed information of the DISQOL Project has been reported elsewhere [14–17]. Five out of 14 DISQOL centers, Edinburgh (United Kingdom), Barcelona (Spain), Prague (Czech Republic), Sicily (Italy), and Tilburg (The Netherlands), collected specific information on PD.

### 2.2. Participants

Three hundred and fifty patients with a diagnosis of idiopathic PD [18], cognitively intact on the regular neurological examination performed at each participating center, and willing to complete the study protocol were included. Ethical approval was obtained from the Local Ethics Committee at each participating center. All patients provided written informed consent prior to their inclusion in the study.

### 2.3. Measures

#### 2.3.1. Satisfaction with Life Scale (SWLS)

The SWLS [8] is a 5-item measure for self-rated assessment of subjective well-being. The questions have a 7-point Likert scale ranging from “strongly disagree” to “strongly agree.” The total score ranges from 5 to 35. A score of 20 represents the neutral point on the scale (the point at which a respondent is neither satisfied nor dissatisfied). Scores are categorized as very high score: highly satisfied (30–35), high score (25–29), average score (20–24), slightly below average in life satisfaction (15–19), dissatisfied (10–14), and extremely dissatisfied (5–9) [8]. Mean life satisfaction scores across samples tend to range from 23 to 28 [19]. The SWLS is reliable, has a high internal consistency, is capable of discriminating groups of presumed different subjective well-being levels, and is efficient and easy to use [8, 9]. Versions (English, Spanish, Czech, Italian, and Dutch) are available at http://internal.psychology.illinois.edu/~ediener/SWLS.html.

#### 2.3.2. World Health Organization Quality of Life (WHOQOL)

The WHOQOL-BREF [20] and WHOQOL-DIS [14] are instruments assessing QoL. The WHOQOL-BREF is a generic QoL questionnaire comprising 24 items covering four domains (physical and psychological health, social relationships, and environment) and two questions about overall QoL (q1. “How would you rate your QoL?”) and satisfaction with health (q2. “How satisfied are you with your health?”). The WHOQOL-DIS is a supplementary module comprising 12 items that function as a single overall domain. In both measures, items have a 5-point Likert-type response format; scores range from 4 to 20, with higher scores representing higher QoL. Both the WHOQOL-BREF and the WHOQOL-DIS modules have the same time frame (i.e., the past two weeks).

#### 2.3.3. Sociodemographic, Health, and Disability Information

In addition, participants provided sociodemographic information on gender, age, marital status, and education, as well as information about their subjective perception of (1) health status: *are you currently ill or in poor health? yes/no*; (2) disability: *do you believe you have a disability? yes/no*; (3) the impact the disability had in life: *how much does this disability affect your life? hardly at all/mildly/moderately/severely/profoundly*. All information was self-reported.

### 2.4. Statistical Analysis

Descriptive statistics were calculated for the demographic variables (i.e., age, gender, marital status, and education), the SWLS items and total score overall QoL, satisfaction with health, self-perception of health (healthy/unhealthy), and disability status (disabled/non-disabled). Floor and ceiling effects would be present if more than 15% of respondents achieved the lowest or highest possible score [21, 22]. Reliability was assessed on the basis of internal consistency (Cronbach's alpha coefficients ≥ 0.70) [23]. Validity analyses were done by construct, concurrent, and discriminant validity approaches. Confirmatory factor analysis (CFA) and known groups validity approaches were used for construct validity testing. CFA was conducted to test whether the original unidimensional structure of the SWLS is suited to Parkinson's disease. The errors of each item were not correlated with each other and the factor variance was set at 1. Goodness of fit was verified by the following fit indices: the comparative fit index (CFI) and the root mean square error of approximation (RMSEA). The models have a satisfactory to good fit when CFI is at least 0.95 [24] and RMSEA values as high as 0.08 are expected to be reasonable in the PD population [25]. Concurrent validity of SWLS was also tested by multiple linear regression analyses by using WHOQOL-BREF's single items: general perceived QoL item (q1. How would you rate your QoL?) and self-rated satisfaction with health item (q2. How satisfied are you with your health?), the WHOQOL-BREF domains (physical health, psychological health, social relationships, and environment), and the WHOQOL-DIS. Effect-size statistics [23] were used for the pairwise comparisons of the ordinal SWLS categories (extremely satisfied to extremely dissatisfied) in regard to WHOQOL domain scores as an alternative way of showing the concurrency of SWLS and WHOQOL.

Age, gender, marital status, and education were used for testing the known groups validity of the SWLS. Student's *t*-tests, one-way analysis of variance (ANOVA), and post hoc Scheffé test were used to examine group differences. Discriminant validity was shown by indicating the difference of SWLS scores between healthy and unhealthy people and disabled and nondisabled people. A *P* value of <0.05 was regarded as statistically significant. All statistical calculations were performed with SPSS for Windows v19.0; CFA was performed with Lisrel version 8.05.

## 3. Results and Discussion

### 3.1. Participants

The mean total SWLS score was 21.1 (SD: 6.7); no significant differences were found on SWLS total scores between centers. However, all centers except Prague had an average total score of 20–24; PD patients from Prague were slightly dissatisfied with life (15–19). Overall mean age was 66.5 (SD = 9.7, range 34–91); patients from Prague were younger than those from Edinburgh and Barcelona (*F*(4, 342) = 3.41, *P* = 0.009). No significant differences were found among gender (Table 1).

Sicily patients had the lowest overall QoL, but significant differences were only found with patients from Edinburgh (*F*(4, 345) = 3.22, *P* = 0.013). Patients with PD from Prague had the lowest satisfaction with health, but significant differences were only found with patients from Edinburgh (*F*(4, 345) = 3.05, *P* = 0.018). Most patients from Sicily and Barcelona reported that they were ill/unhealthy (*χ* ^2^(4) = 34.61, *P* < 0.001). About 90% or more of the participants reported being disabled, with the exception of Barcelona (67.7%) (*χ* ^2^(4) = 24.02, *P* < 0.001) (Table 1).

### 3.2. Descriptive Statistics

No items showed floor or ceiling effects; we found 0.9% at floor and 0.3% at ceiling. Missing information varied from 1.9% (item 3) to 2.5% item 1.

### 3.3. Reliability

**Figure 1:**
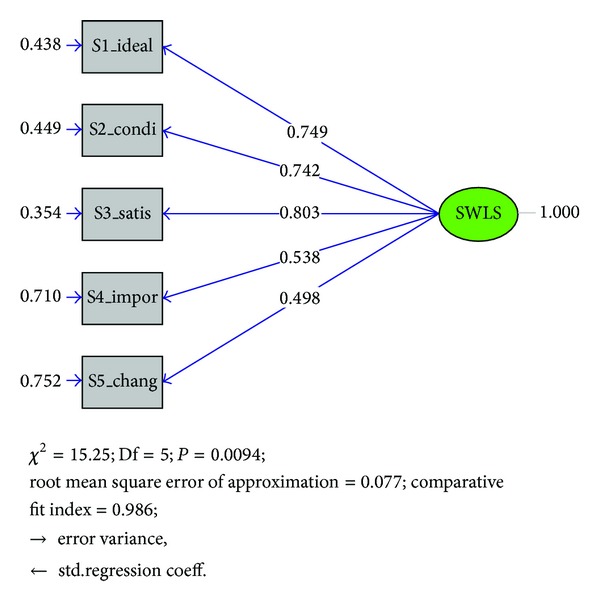
Confirmatory factor analysis results for one factor 5-item SWLS.

Cronbach's alpha coefficient was 0.81 for the total sample and varied between centers, from 0.74 (Barcelona) to 0.88 (Tilburg). Deleting items would not result in improvement in the internal consistency. The *χ* ^2^ value of this model was 15.25 (df = 5, *P* = 0.00936). The CFA results indicated acceptable scale structure via quite satisfactory fit indices (CFI = 0.99; RMSEA = 0.077); error variance was also found acceptable (between 0.20 and 0.85) for each of the items (Figure 1).

### 3.4. Discriminant Validity

In relation to known groups, PD patients younger than 65 scored significantly lower than those 65 and older (*t*(345) = −3.398, *P* = 0.001). No differences were found for gender, marital status, or education (Table 2).

The SWLS was able to discriminate between healthy and unhealthy participants (*t*(346) = 4.52, *P* < 0.001), disabled and nondisabled (*t*(346) = 2.72, *P* = 0.007), and those with mild versus severe impact of the disability on their lives (*t*(308) = 5.36, *P* < 0.001). Healthy participants, nondisabled, and those reporting a mild effect of the disability on their lives scored significantly higher on life satisfaction than ill/unhealthy, disabled, and participants reporting a severe impact of the disability on their lives. Yet, only ill/unhealthy and those reporting a severe impact of the disability on their lives fell in the category of *slightly dissatisfied*. In addition, the SWLS scores decreased as impact disability in life increased; SWLS scores were 27.86 (SD = 6.33) for those reporting no impact (hardly at all), 24.44 (SD = 6.15) for those reporting a mild impact, 21.21 (SD = 6.00) for those reporting a moderate impact, and 19.47 (SD = 6.06) and 17.21 (SD = 6.85) for those reporting severe and profound impact, respectively (*F*(4, 305) = 13.73, *P* < 0.001).

### 3.5. Concurrent Validity

The two independent questions of the WHOQOL (q1. How would you rate your QoL? and q2. How satisfied are you with your health?), the four domains of the WHOQOL-BREF (physical health, psychological health, social relationships, and environment) and the WHOQOL-DIS were used in the analyses of concurrent validity of SWLS as shown in the Table 3. All of the regression models (models 8–14) in which total SWLS score is compared with WHOQOL-BREF's overall QoL (q1), health satisfaction (q2) items, the four domains, and the WHOQOL-DIS showed very satisfactory standardized beta values, indicating that the variances of WHOQOL could be sufficiently explained by the overall SWLS score. On the other hand, models 1–7 were conducted to see the concurrence of the individual items of the SWLS on the WHOQOL-BREF and the WHOQOL-DIS. All of the items were found very sensitive to all of the WHOQOL-BREF domains. The physical domain of the WHOQOL was not sensitive to the 3rd item of the SWLS (satisfied with life). It was also interesting to find that the variance of the environmental domain was significantly explained by all SWLS items except for the 1st item (Life close to ideal). Satisfactory effect sizes (greater than 0.2) were obtained for almost all of the adjacent SWLS categories (Table 4).

## 4. Discussion

The aim of this study was to explore the psychometric properties of the SWLS in people with PD and assess variables associated with SWLS across five European centers. The SWLS is a useful tool to be used with other measures to provide valuable information throughout the continuum of care on people with PD, from early diagnosis to late phases of the disease; the information obtained might serve to guide the evaluation and clinical decision making of professionals, the effectiveness of care, and service delivery, in both health and social care. Also, the information might prove useful for policymakers; improvement of welfare systems, transportation, housing, access to leisure activities, and adaptation of working environment regulations [15] are nonclinical aspects which might contribute to a better degree of wellbeing and life satisfaction.

In terms of reliability, SWLS showed acceptable levels of internal consistency for the total sample (0.81) at each participating center (range 0.74 to 0.88). Although there is, in the literature, a considerable degree of variability across samples, generally, the levels of internal consistency are around 0.80 [19]. Our findings are quite similar and comparable to those reported previously in clinical samples [11–13] and population based studies [26–28]. CFA confirmed that a single-factor solution model reveals an adequate fit on the basis of model fit indices (RMSEA and CFI). This result also supported previous findings reported from factor analyses [12, 13, 26–32] and confirmed the hypothesized factor structure for the SWLS [8]. In our sample, items 4 and 5 had weaker association with satisfaction of life than items 1–3 consistent with findings reported in previous studies [27, 30].

In addition to factorial structure, the SWLS was found to have good concurrent and discriminant validity. Previous studies using the SWLS with QoL and health-related QoL measures reported moderate-high correlations between both constructs. For example, statistically significant positive correlations were reported between the SWLS and all SF-36 domains in patients with systemic lupus erythematous [11] and among the SWLS, the WHOQOL-BREF domains, and WHOQOL-DIS in patients with multiple sclerosis [13]. Also, statistically significant negative correlations were found between the SWLS and the Hospital Anxiety and Depression Scale in people with MS [13].

In relation to the discriminant validity, patients with systemic lupus erythematous [11] and patients with multiple sclerosis reporting a more severe effect of disability on their life [13] had lower scores in both health-related quality of life and satisfaction with life.

The SWLS was able to discriminate between participants on the basis of their health perception (healthy versus unhealthy), disability status (disabled versus nondisabled) and impact of disability on their lives. Although SWLS scores were higher for healthy, nondisabled, and those reporting less impact of the disability in their life, only the former and the last groups scored slightly below average in life satisfaction (15–19). Patients with systematic lupus erythematous [11], arthritis [12], and multiple sclerosis [13] and people in the general population with a mental diagnosis in the previous 12 months [10] also scored below average in life satisfaction. However, none of these studies reported information in relation to the percentage of participants being extremely dissatisfied, dissatisfied, average, or satisfied. This information is important because depending on the source of the dissatisfaction, measures might exist that can be taken to improve life satisfaction in clinical populations.

Pairwise comparisons of the 1st versus 2nd (extremely dissatisfied versus dissatisfied) and 5th versus 6th (slightly satisfied versus extremely/highly satisfied) SWLS categories in regard to WHOQOL-BREF domain scores yielded meaningful (>0.50) effect-size figures compared to those of comparisons of the middle descriptors (i.e., 2nd, 3rd, 4th, and 5th descriptors). Physical and psychological domains of the WHOQOL-BREF can better discriminate the 1st versus 2nd categories than the remaining two domains of the WHOQOL-BREF (see Table 4). These findings may be evidence of nonlinear association between life satisfaction and quality of life in the PD patients.

We did not find a linear pattern of lower life satisfaction with increasing years of disability; however, we found that life satisfaction decreased as the impact of disability in life increased. Whereas it has been reported that PD patients were generally satisfied with the care they received, especially that from movement disorder specialists [33], it has been also suggested that “improving recognition of the period of preclinical disability will enable better timing of therapies to delay the onset of disability in PD” [34].

People with disabilities experience more barriers with access to health and social services as well as social and other environmental barriers [13, 15, 17] than the general population. Thus, barriers might be a source of life dissatisfaction in PD patients. From a social point of view and social policies, this information is important because in some cases, PD patients with disabilities might have further benefits in life improvement and life satisfaction from social and environmental policies apart from health policies. For example, implementation of social and environmental policies related to access to public transportation and availability of special transport when needed, ramps; easy access to places for leisure activities (e.g., cinemas, theaters, and restaurants); support for going on vacation; financial support from government, which have been shown to be important for QoL of PD patients [15, 17], might increase as well their life satisfaction. This might be reflected on the association found between the SWLS and the environmental domain of the WHOQOL-BREF which includes items related to health care access, transport, environment, and financial resources.

Compared to studies which assessed life satisfaction with the SWLS in elderly people [27, 32], we found lower SWLS scores in PD patients in all participating centers. Our results reveal that PD has a great influence on the patients' quality of life as well as their satisfaction with life. Overall, these findings not only confirm the relationship between life satisfaction and QoL, but also show the distinctiveness between them, suggesting that QoL and life satisfaction may be assessed separately to enable a full examination of the patient's state [11].

Study limitations include the following: (1) we used a targeted population; (2) the information which usually characterizes PD patients, as Hoehn-Yahr scores and UPDRS scores, was not recorded in all centers; (3) patients with mild cognitive impairment, commonly found in people with PD, were not included; (4) we did not examine the test-retest reliability. Despite these limitations, in the light of our findings, the SWLS was found to be acceptable and reliable and shows evidence of validity in patients with PD.

## 5. Conclusion

In conclusion, the results revealed that the psychometric properties of the SWLS were satisfactory. In addition, the results confirmed that the SWLS is suitable to use cross-culturally in patients with PD. The use of the SWLS incorporated during routine visits and in future longitudinal studies will help to determine its sensitivity over time to disease progression and disability and to different therapeutic interventions (pharmacological, psychological, physiotherapy, and surgical) or implementation of different health and social service provision or improvement in environmental policies. To our knowledge, this is the first study using the SWLS cross-culturally in people with PD in several European countries, thus, adding evidence of its validity in a population who was clearly warranted [11].

## Figures and Tables

**Table 1:** SWLS scores, age, and other health variables by centre.

	Edinburgh *N* = 123	Barcelona *N* = 65	Prague *N* = 32	Sicily *N* = 26	Tilburg *N* = 104	Test_df*_, sig
SWLS: mean (SD)	21.1 (6.1)	21.3 (6.2)	18.5 (7.1)	21.2 (7.0)	21.8 (7.5)	*F*_(4,345)_ 1.469, 0.211
Age years: mean (SD)	67.1 (8.5)	68.8 (9.9)	61.6 (8.8)	64.6 (10.7)	66.7 (10.2)	*F*_(4,342)_ 3.414, 0.009
Male: *n* (%)	63 (51.2)	34 (52.3)	21 (65.6)	18 (69.2)	60 (57.7)	*χ*_(4)_^2^ 4.672, 0.323
Married: *n* (%)	90 (73.2)	48 (73.8)	28 (90.3)	24 (93.2)	86 (84.3)	*χ*_(4)_^2^ 10.608, 0.031
College and university education: *n* (%)	53 (43.1)	16 (24.6)	16 (50.0)	4 (15.4)	39 (37.5)	*χ*_(4)_^2^ 13.816, 0.008
Overall QOL^1^: mean (SD)	3.5 (0.9)	3.3 (0.8)	3.4 (0.6)	2.9 (1.0)	3.3 (0.9)	*F*_(4,345)_ 3.220, 0.013
Satisfaction with health^2^: mean (SD)	2.9 (1.0)	2.8 (1.0)	2.2 (0.8)	2.8 (1.0)	2.7 (0.9)	*F*_(4,345)_ 3.047, 0.018
Ill health/unhealthy: *n* (%)	53 (43.4)	52 (80.0)	17 (53.1)	23 (88.5)	54 (52.4)	*χ*_(4)_^2^ 34.606, <0.001
Disabled: *n* (%)	108 (87.8)	44 (67.7)	31 (97.0)	27 (90.0)	95 (93.1)	*χ*_(4)_^2^ 24.020, <0.001

^1^Overall QoL (q1). “How would you rate your QoL?.”

^2^Satisfaction with health (q2). “How satisfied are you with your health?.”

*df: degrees of freedom.

**Table 2:** Known groups comparisons and discriminant validity results. *n* = 350.

	SWLS: score mean (sd^1^)	*t*-test, df^2^; *P* value
*Known groups *		
Gender		
Male	21.6 (7.0)	1.680, 348; 0.089
Female	20.4 (6.2)	
Group age		
<65 years	19.5 (6.7)	−3.398, 345; 0.001
≥65 years	22.0 (6.5)	
Marital status: *n* (%)		
Other	20.0 (6.5)	−1.500, 345; 0.135
Married	21.4 (6.7)	
Education: *n* (%)		
Primary school or less	20.3 (6.6)	−0.924, 346; 0.356
Secondary school and higher	21.2 (6.7)	
*Discriminant validity comparisons *		
Self-reported health		
Healthy	23.0 (6.5)	4.517, 346; <0.001
Ill/unhealthy	19.8 (6.5)	
Self-reported disability		
No	23.5 (6.3)	2.717, 346; 0.007
Yes	20.7 (6.6)	
Disability effect in life		
Mildly and moderately	22.7 (6.4)	5.363, 308; <0.001
Severely and profoundly	18.6 (6.3)	

^1^sd: standard deviation, ^2^df: degrees of freedom.

**Table 3:** Concurrent validity 1: multiple linear regression models' results for SWLS-dependent variables are WHOQOL general perceived quality of life (q1), self-rated health (q2), four WHOQOL-BREF dimensions (physical, psychological, social relationships, and environmental well-being), and WHOQOL disability module.

	Dependent variables						
	Overall QoL^1^	Self-rated health^2^	Physical health (WHOQOL)	Psychological health (WHOQOL)	Social relationships (WHOQOL)	Environment (WHOQOL)	WHOQOL disability module
	Model 1	Model 2	Model 3	Model 4	Model 5	Model 6	Model 7
	Beta	Beta	Beta	Beta	Beta	Beta	
(Constant)	*P* < 0.001	*P* = 0.01	*P* < 0.001	*P* < 0.001	*P* < 0.001	*P* < 0.001	*P* < 0.001
Age	−0.05	0.02	−0.002	−0.12*	−0.10*	−0.10*	−0.19**
S1 In most ways my life is close to my ideal	0.03	0.05	0.11	0.07	0.05	−0.04	0.02
S2 The conditions of my life are excellent	0.20**	0.08	0.12	0.02	−0.001	0.21**	0.12*
S3 I am satisfied with my life	0.36**	0.37**	0.12	0.35**	0.24**	0.18**	0.15*
S4 So far I have gotten the important things I want in life	0.02	0.04	0.14*	0.07	0.17**	0.24**	0.09
S5 If I could live my life over, I would change almost nothing	−0.04	0.07	0.06	0.08	0.12*	0.11*	0.16**

	Model 8	Model 9	Model 10	Model 11	Model 12	Model 13	Model 14

(Constant)	*P* < 0.001	*P* < 0.001	*P* < 0.001	*P* < 0.001	*P* < 0.001	*P* < 0.001	*P* < 0.001
Age	−0.073	0.01	−0.01	−0.13*	−0.11*	−0.10*	−0.18**
Total SWLS	0.46**	0.48**	0.41**	0.46**	0.43**	0.52**	0.40**

*P < 0.05.

**P < 0.001.

^1^Overall QoL (q1). “How would you rate your QoL?.”

^2^Satisfaction with health (q2). “How satisfied are you with your health?.”

**Table 4:** Concurrent validity 2: pairwise comparisons of the SWLS categories in regard to WHOQOL domain scores.

	Comparison of the SWLS categories*					
WHOQOL domains	1 versus 2 ES**	2 versus 3	3 versus 4	4 versus 5	5 versus 6	1 versus 6
Physical health	0.69	0.39	0.13	0.25	0.63	1.63
Mental health	0.62	0.29	0.25	0.34	0.75	1.69
Social relationships	0.03	0.38	0.21	0.44	0.56	1.34
Environmental	0.19	0.53	0.12	0.30	0.83	1.90

*1: extremely dissatisfied; 2: dissatisfied; 3: slightly dissatisfied; 4: neutral/average; 5: slightly satisfied; 6: extremely/highly satisfied.

**ES (Cohen's effect size): difference between means divided by pooled standard deviation; interpretation: 0.2: small, 0.5: medium, 0.8: large.
